# 

**Published:** 1871-12

**Authors:** 


### TABLE OF CONTENTS.

### ORIGINAL COMMUNICATIONS.

THE CAUSATION OF DISEASE—CONTAGION. By FRANK A. RAMSEY, M.D., Knoxville, Tenn., Vice-President of the Tennessee Medical Society; President of the Medical Society of East Tennessee, etc. .... 491

A NEW INSTRUMENT FOR TREATING LOCALLY CERTAIN DISEASES OF THE GENITO-URINARY ORGANS, MALE OR FEMALE. By WM. ABRAM LOVE, M.D., Atlanta, Ga. .... 506

THE HYGIENE OF GESTATION. (Abstract of a Paper Read before the Atlanta Academy of Medicine, being a part of the Report of the Section on Hygiene.) By JOHN STAINBACK WILSON, M.D., Atlanta, Ga. .... 512

ATLANTA ACADEMY OF MEDICINE. J. T. JOHNSON, M.D., Reporter .... 526

ABSTRACTS AND CONDENSATIONS FROM GERMAN JOURNALS. By CH. RAUSHENBERG, M.D., Atlanta, Ga. .... 533

### EDITORIAL AND MISCELLANEOUS.

A NEW SPIRIT IN THE MEDICAL PROFESSION OF GEORGIA .. 544

FOREIGN CORRESPONDENCE .... 545

DEATH OF A PROMINENT PHYSICIAN FROM THE HYPODERMIC USE OF MORPHINE .... 546

CUNDURANGO A FAILURE .... 548

BIBLIOGRAPHICAL .... 549

